# Psychological impact of COVID-19 pandemic: Protocol and results of first three weeks from an international cross-section survey - focus on health professionals

**DOI:** 10.1016/j.jadr.2020.100005

**Published:** 2020-12

**Authors:** Shanaya Rathod, Saseendran Pallikadavath, Allan H. Young, Lizi Graves, Mohammad Mahbubur Rahman, Ashlea Brooks, Mustafa Soomro, Pranay Rathod, Peter Phiri

**Affiliations:** a Research department, Southern Health NHS Foundation Trust, UK; b Portsmouth-Brawijaya Centre for Global Health, Population, and Policy University of Portsmouth, UK; c Kings College London, UK; d Solent NHS Trust, St Marys community Mental health campus, Portsmouth, UK; e PPI Rep, London, UK

**Keywords:** Covid-19, Healthcare professionals, Psychological impact

## Abstract

**Background:**

The psychological impact of COVID-19, resultant measures and future consequences to life will be unveiled in time.

**Aim:**

To investigate the psychological impact of COVID-19, resultant restrictions, impact on behaviours and mental wellbeing globally. This early analysis, explores positive and adverse factors and behaviours with focus on healthcare professionals.

**Methods:**

This is a cross-sectional survey, using a questionnaire based on published approaches to understand the psychological impact of COVID-19. The survey will be repeated at 6 months because of rapidly changing situation.

**Results:**

We have presented results from first 3 weeks of the survey. Conclusions may change as more individuals take part over time. 7,917 participants completed the survey in the first 3 weeks; 7,271 are from the United Kingdom. 49.7% of the participants are healthcare professionals. There is high representation of female participants. Participants reporting suicidal thoughts is 32%. Healthcare professionals have reported mild depression and anxiety in higher proportions. Increasing age and female gender report higher compliance with government advice on COVID 19 whereas higher education, homeowners, key worker status, high alcohol, drug use and participants with pre-existing suicidal thoughts reported low compliance with government advice. Participants who reported suicidal thoughts pre-COVID are less likely to communicate with friends and family, or engage in coping strategies.

**Conclusions:**

Evidence has shown an adverse psychological impact of previous pandemics on the population, especially wellbeing of healthcare professionals. Research should focus on identifying the need, preparing services and determining the factors that enhance and build resilience.

**Funding:**

This survey is linked to a MRC global health research program of the Portsmouth-Brawijaya center for Global Health, Population, and Policy, (MR/N006267/1), University of Portsmouth.

## Highlights

• Understanding the psychological impact of the COVID-19 pandemic will be vital for service planning and delivery.
• Initial results in UK show healthcare professionals report more mild anxiety and depression than non-healthcare professionals.
• Increasing age and female gender are associated with higher compliance with government guidelines.
• Participants who report suicidal thoughts pre-COVID are less likely to communicate with friends and family, or engage in coping strategies.

## 1 Background

The COVID-19 pandemic has threatened the health and lives of millions of people across the globe. On 30th January 2020, the World Health Organization declared a public health emergency of international concern, and governments were urged to prepare for the global spread of COVID-19 from East Asia (World Health Organisation, 2020). The United Kingdom (UK) government and its advisers initially had a four-pronged plan (of 3rd March 2020) to contain, delay, research, and mitigate (Department of Health and Social Care, 2020). Subsequently, on 23rd March 2020, the UK went into lockdown. In early May, there was some easing for hardware and home improvement stores, and on 11th May 2020, ‘the plan to rebuild’ in England was published.

While the primary focus has been on preventing transmission of the virus, finding vaccines and a cure, there is a realization that the effects and aftermath of this crisis, especially for mental health globally, could be unprecedented. These may range from the understandable anxiety related to health, life and global uncertainty (Yao et al., 2020), to the effects of restrictions that have been placed on lives in the form of social distancing (Bedford et al., 2020), self-isolation and quarantine regimes (Reynolds et al., 2007; Memish et al., 2020). A recent review reported negative psychological effects, including post-traumatic stress symptoms, confusion, and anger. Stressors reported in quarantine included longer quarantine duration, infection fears, frustration, boredom, inadequate supplies, inadequate information, financial loss and stigma (Brooks et al., 2020). Vindegaarda, and Benros (2020) completed a systematic review of 43 studies measuring psychiatric impact associated with COVID-19 and concluded that further research is needed for preventive measures during potential subsequent pandemics.

Key workers, younger adults, those living in over-crowded households, and individuals with health conditions (especially mental health conditions) have reported more daily stressors (Fancourt et al., 2020). Changes in behavior and adaptations determine perceived levels of stress, depression and anxiety. Emerging evidence suggests that individuals with pre-existing psychiatric disorders have experienced worsening of psychiatric symptoms (Vindegaard and Eriksen Benros, 2020).

Given this unpreceded situation, health and social care workers on the frontline are directly involved in the treatment and care of patients with COVID-19, which has led to an overwhelming workload. The working environment and lack of personal protection equipment (PPE) emphasize the need to investigate the psychological impacts of COVID-19 on health and social care workers. Recent studies investigating health care workers found increased depression/depressive symptoms, anxiety, psychological distress, and poor sleep quality (Vindegaard and Eriksen Benros, 2020; Lai et al., 2020). Previous studies on the outbreaks of other infectious diseases, such as severe acute respiratory syndrome (SARS) and Middle East respiratory syndrome (MERS), have consistently showed adverse psychological impacts on health care workers. These impacts include a high level of anxiety and depression and stress that resulted in meeting the diagnosis of post-traumatic stress disorder (Lee et al., 2018; Tam et al., 2004). Other studies have shown that the potential to transmit the disease to families and friends have been a fear weighing in healthcare professionals minds (Rubin et al. 2020; Wingfield et al. 2020; Brooks et al., 2020).

Despite the rapidly building evidence on the impact of COVID 19, there are significant gaps due to the unprecedented nature of the pandemic and the resultant changes across the globe. Each study makes a unique contribution and adds a different perspective, thereby improving generalisability and our understanding of the landscape.

Given the unique situation we face, we sought to explore, via an international study, the psychological impact of COVID-19, the resultant restrictions and impact on emotions, behaviours and changes in mental health and wellbeing.

### 1.1 Main purpose of this study

To investigate the psychological impact of COVID-19, the resultant restrictions and impact on behaviours and changes in mental wellbeing across the global population. We also aim to explore what pre-COVID-19 factors and behaviours may support people's wellbeing and what might have a negative impact.

The study is designed to explore the psychological impact of COVID-19 on the following groups though they are not mutually exclusive:

• General population
• Individuals with pre-existing vulnerabilities such as mental health conditions
• Individuals with families of COVID-19
• Healthcare professionals (HCP)

For interim analysis in first three weeks of the study we wanted to investigate:

1 Are families with experience of COVID-19, healthcare workers, and people with pre-existing mental health conditions or other co morbid conditions or vulnerabilities more likely to experience mental health consequences compared to others?
2 Are there differences in psychological impact of COVID 19 for different demographics

Null hypothesis: There will be no difference in the psychological impact of COVID-19 between the different groups or by demographics.

## 2 Methodology

This is a repeated, cross-sectional study. The survey will be repeated at 6 months because of rapidly changing situation and potential of second wave of the pandemic, predicted in the winter. We devised an online questionnaire, based on published approaches, to understand the psychological impact of COVID-19 and the resultant restrictions. Five standardised measures have been included to explore levels of depression (Patient Health Questionnaire-9; Kroenke and Spitzer, 2002), anxiety (GAD-7; Generalised Anxiety Disorder Assessment; Spitzer et al., 2006) impact (The Impact of Event Scale- Revised; IES –R; Weiss and Marmar, 1997), loneliness (a brief loneliness scale, Hughes et al., 2004) and social support (The Multidimensional Scale of Perceived Social support; MSPSS, Zimet et al., 1988)

The questionnaire has several versions. The first version has been implemented in the UK, and the other versions have been adapted based on cultures and landscapes of different countries. The adaptations are culturally informed and translated into local languages to make them relevant and sensitive to local populations. The questions have been investigated for face and content validity with a limited relevant sample before using them in the survey.

We have reviewed other questionnaires currently being implemented and found that each is unique and different in what it is trying to measure. For an unknown entity like COVID-19, over-inclusiveness and repetition improves validity.

In the UK, the questionnaire was implemented on 1st May 2020 with Southern Health NHS Foundation Trust as the sponsor. Since then, 50 National Health Service (NHS) Trusts, Universities and The center for Applied Research and Evaluation International Foundation (CAREIF) have collaborated and are advertising the survey to their staff, patients and public. Organisations joined slowly as in the first week there were 18 organisations, 37 in the second week and 42 in the third week of this analysis paper. Of the 42 NHS organisations, 26 were mental health and community trusts and 18 were acute NHS organisations. Further organisations joined and recruitment continues until 31st July 2020 for the first wave. The second wave will commence from 1st October to 31st December 2020.

## 3 Study sample

This is a participatory study, as the sampling is based on self-selection by the participants if they meet the criteria.

### 3.1 Inclusion criteria

The inclusion criteria for this survey are broad to capture the views of any individual above 16 years of age who would like to respond to the online questionnaire. We aim to include members of the public, keyworkers, including HCP, individuals who have suffered COVID-19, and those with vulnerabilities like Diabetes, Hypertension, pre-existing mental health conditions.

### 3.2 Exclusion criteria

No specific exclusion criteria apart from those unwilling or who lack the capacity to participate.

## 4 Recruitment

Participants are invited to participate in the study via multiple media sources, including social media, newsletters, communication platforms within participating organisations and countries.

Informed consent is implied. Participants are allowed as much time as they wish to consider the information and to decide whether to participate in the study. The survey includes a participant information sheet detailing the relevant information regarding the study. Participants have the right to withdraw from participating in the study before submitting it. The surveys are anonymous, participants can leave an email address if they would like to be contacted about the second wave of the study and this is stored separately to their survey data.

## 5 Questionnaire design

The questionnaire is designed to be completed using Qualtrics XM, a cloud-based online platform. The survey consists of 35 questions divided into 5 parts. It takes 12–16 min to complete. The different sections of the questionnaire are:

PART A: Demographics - this section ascertains participant characteristics such as age, gender, ethnicity, nationality, and country in which they reside, religion, level of education, employment status and sector and accommodation.

PART B: About participants’ health including core questions about health and wellbeing.

PART C: Participants’ experience and knowledge of COVID-19 including access to COVID-19 information guidance and updates, compliance to advice, information guidance and updates, changes in behaviours, including self-isolation.

PART D: Psychological impact including validated measures adapted for this survey. These are PHQ-9 (Patient Health Questionnaire-9; Kroenke and Spitzer, 2002), GAD-7 (Generalised Anxiety Disorder Assessment; Spitzer et al., 2006) and the Impact of Event Scale- Revised (IES –R; Weiss and Marmar, 1997).

PART E: Ways of coping, exploring what changes in behavior and social contacts are made to cope with any restrictions on lifestyle; a brief loneliness scale (Hughes et al., 2004) and the Multidimensional Scale of Perceived Social support (MSPSS) (Zimet et al., 1988).

## 6 Study analysis strategy

We aim to provide timely information to inform the public, organisations, and policymakers regarding the psychological impact of COVID-19. Therefore the following analytic strategy is used for this paper that focusses on rapid response, initially with three weeks data. As a high proportion of respondents in our dataset is HCP, we have focussed our interim analysis on this group.

Descriptive statistics for categorical data are presented as frequencies and proportions for the whole sample and relevant subgroups. The association between categorical variables is considered through *t*-test. Statistical significance is indicated by p-values.

Outcomes are measured as categorical variables. Except for the outcome ‘change in suicidal thoughts due to corona virus’, that has two categories (yes/no), all other outcomes have more than two categories, and those multiple categories are ordinal (e.g., GAD-7: 0–4 score implies minimal anxiety, 5–9 mild anxiety, 10–14 moderate anxiety, and 15–21 severe anxiety) variables. For all outcomes, we have conducted ordered logit regressions with adjustment for appropriate (observed) confounders. We have reported changes in log-odds ratios as coefficients, and marginal effects of healthcare professional dummy, and those by their gender. The statistical software packages SPSS and R are employed for data and regression analyses as appropriate.

Missing data figures indicating a selection bias, are high for few questions but generally there has been good response to most questions. Therefore we did not conduct tests like instrumental variable regression.

## 7 Data management

We aim to present the results of this study in aggregate form, with no individuals being identified. All data is being collected in a secure password protected Qualtrics XM online cloud based platform. Access to systems is restricted to specific individuals whose access is monitored and audited for compliance.

Data exported from the survey platform is anonymous, stored and managed in password protected files on encrypted computers and servers. Access to electronic data is limited only to members of the research team. Study documentation will be archived in accordance with guidelines for Good Clinical Practice and in NHS approved, secure and adequate archiving facility. Research personnel will keep information relevant to the study for up to 15 years, and then will be destroyed.

## 8 Ethics

The study received ethics and HRA approval (IRAS project ID: 282,858; REC reference: 20/HRA/1934) on 27 April 2020.

## 9 Results

### 9.1 Summary statistics

7917 participants completed the survey in the first three weeks. Of those, 7271 individuals are from the United Kingdom. All participants did not respond to all questions, and hence, there are some missing values as already discussed and presented in the tables.

### 9.2 Part A: demographics

Among 7917 participants, 3933 (49.7%) identified themselves as HCP. Of the UK participants, approx. 52% identified themselves as HCP. Among the non-HCP, approximately 6% receive mental health services and approx. 43% are from the general population as per the categories of participants we sought. As this is initial data the numbers in different categories are variable. Table 1 shows summary statistics of demographics by non-HCP and HCP that are the large groups.

**Table 1:** Summary statistics of demographics by healthcare professional.

		Non-healthcare Professional (*N* = 3984)		Healthcare Professional (*N* = 3933)		Mean Diff.	p-value
	*N*	Mean	SD	Mean	SD		
Age group (*N*=7513; missing=404)							
Under 21	154	0.039	0.192	0.004	0.064	−0.034	0.000
21–24	363	0.057	0.231	0.041	0.198	−0.016	0.001
25–34	1503	0.185	0.388	0.214	0.410	0.029	0.001
35–44	1753	0.207	0.405	0.258	0.437	0.051	0.000
45–54	1864	0.207	0.405	0.286	0.452	0.079	0.000
55–64	1406	0.193	0.395	0.182	0.386	−0.011	0.208
65 and over	470	0.114	0.317	0.016	0.126	−0.098	0.000
Ethnicity (*N*=7528; missing=389)							
White	6821	0.922	0.268	0.891	0.311	−0.031	0.000
Asian	237	0.022	0.146	0.040	0.197	0.019	0.000
Black	73	0.007	0.081	0.012	0.111	0.006	0.010
Others	397	0.049	0.217	0.056	0.229	0.006	0.225
Religion (*N*=7448; missing=469)							
Christian	3382	0.447	0.497	0.460	0.498	0.013	0.252
Muslim	60	0.006	0.076	0.010	0.100	0.004	0.044
Sikh	16	0.002	0.041	0.003	0.051	0.001	0.404
Hindu	80	0.006	0.080	0.015	0.120	0.008	0.001
Jewish	35	0.005	0.073	0.004	0.064	−0.001	0.450
Buddhist	55	0.006	0.080	0.008	0.090	0.002	0.363
No religion	3630	0.500	0.500	0.476	0.499	−0.023	0.043
Others	190	0.027	0.163	0.024	0.152	−0.004	0.306
Gender (*N*=7483; missing=434)							
Male	1251	0.213	0.409	0.126	0.331	−0.087	0.000
Female	6232	0.787	0.409	0.875	0.331	0.087	0.000
Left Education (*N*=7507; missing=410)							
Before age 16	143	0.029	0.167	0.010	0.100	−0.019	0.000
At age 16	970	0.175	0.380	0.087	0.282	−0.088	0.000
At age 18	1194	0.207	0.405	0.115	0.319	−0.092	0.000
Attended University	4713	0.509	0.500	0.736	0.441	0.227	0.000
Prefer not to say	66	0.012	0.110	0.006	0.075	−0.007	0.002
Others	421	0.067	0.251	0.046	0.209	−0.022	0.000
Accommodation type (*N*=7515; missing=402)							
Own home	5409	0.694	0.461	0.743	0.437	0.049	0.000
Shared accommodation	143	0.018	0.133	0.020	0.140	0.002	0.576
Private rented accommodation	1144	0.145	0.352	0.159	0.366	0.015	0.077
Halls of Residence	2	0.001	0.024	0.000	0.000	−0.001	0.139
Parent's home	594	0.102	0.303	0.058	0.233	−0.044	0.000
Other	223	0.040	0.197	0.020	0.140	−0.021	0.000
Key worker (*N*=6114; missing=1803)							
No	1129	0.401	0.490	0.044	0.204	−0.357	0.000
Health and Social care	4302	0.330	0.470	0.947	0.223	0.617	0.000
Education and Childcare	277	0.110	0.313	0.003	0.054	−0.107	0.000
Key public services	65	0.026	0.158	0.001	0.028	−0.025	0.000
Local and national government	115	0.045	0.208	0.002	0.040	−0.044	0.000
Food and other necessary goods	77	0.031	0.174	0.001	0.023	−0.031	0.000
Public safety and national security	15	0.006	0.079	0.000	0.000	−0.006	0.000
Transport	33	0.013	0.114	0.000	0.016	−0.013	0.000
Utilities, Communication and financial	72	0.028	0.164	0.001	0.037	−0.026	0.000
Prefer not to say	29	0.010	0.097	0.002	0.040	−0.008	0.000

*Note:* Mean is a proportion of individual in a category. If we multiply means by 100, we will get percentages. SD is standard deviation.

Means in age categories imply that participating HCP are proportionately higher in middle age groups than non-HCP. Professionals from White ethnic background are 3.1% lower in the sample (with statistical significance) in the HCP group than the non-HCP group; indicating higher proportions of Asian and Black ethnic minority professionals in the health sector than within the participating population. Muslims and Hindus present significantly higher in HCP, but individuals with no stated religion mostly work in the non-healthcare sector. The data shows a higher proportion of females in the healthcare profession compared to their male counterparts. Higher education is reported in higher proportions in the healthcare professions, as the data suggests 22.7% more HCP have attended University education compared to non-healthcare workers.

In the sample, around 5% more HCP live in their own houses compared to non-HCP. Halls of residence, parent's home and other accommodation show statistically significant negative differences in means, implying lower proportions of the participating HCP live in this type of accommodation. The response rate on key worker status is low due to missing values. Among 4302 respondents who identified as keyworkers, most work in the health and social care sector, with only 369 in the social care sector. Among non-HCP, 33% work in the health and social care sector, implying that many respondents work in this sector, but they do not consider themselves as HCP.

### 9.3 Part B: about your health

Table 2 shows the summary statistics of pre-COVID health condition and wellbeing. Most of the participants (4661) have not reported pre-existing listed health problems. In theory, a person might have multiple health conditions, but in this data, we did not see that. The largest pre-existing condition reported is depression. Social phobia, Post-Traumatic Stress Disorder, Alcohol/Drug problems, Bi-polar disorder, and Personality disorder show to be statistically and significantly lower in HCP than others, except for Anorexia. Participants who reported drinking alcohol 4 times or more a week or never are significantly lower among HCP. Drug users present as lower in the HCP group, although overall numbers are low. 2309 (32%) participants reported experiencing suicidal thoughts, with no statistical differences between the groups. Non-HCP seek help from counsellors/psychologist/psychotherapist and Community Mental Health Teams in higher proportions compared to HCP according to the data.

**Table 2:** Summary statistics of pre-existing health conditions and others by healthcare professional.

		Non-healthcare Professional (*N* = 3984)(		Healthcare Professional (*N* = 3933)		Mean Diff.	p-value
	*N*	Mean	SD	Mean	SD		
Pre-existing health condition (*N*=7132; missing=785)							
Anxiety	499	0.070	0.255	0.070	0.256	0.001	0.929
Panic Attacks	126	0.020	0.140	0.016	0.124	−0.004	0.154
Anorexia	10	0.001	0.024	0.002	0.046	0.002	0.079
Psychosis	4	0.001	0.030	0.000	0.016	−0.001	0.274
Depression	1206	0.168	0.374	0.170	0.375	0.001	0.894
Bulimia	29	0.004	0.064	0.004	0.063	0.000	0.949
Social phobia	65	0.012	0.110	0.006	0.078	−0.006	0.006
Attention deficit disorder	25	0.004	0.066	0.003	0.052	−0.002	0.217
Obsessive Compulsive Disorder	108	0.014	0.116	0.017	0.128	0.003	0.286
Post-Traumatic Stress Disorder	218	0.036	0.187	0.025	0.157	−0.011	0.006
Alcohol/Drug problems	14	0.003	0.054	0.001	0.033	−0.002	0.075
Bi-polar disorder	59	0.010	0.101	0.006	0.080	−0.004	0.072
Personality disorder	108	0.021	0.143	0.010	0.099	−0.011	0.000
None of the above	4661	0.635	0.481	0.670	0.470	0.035	0.002
Frequency of drinking alcohol (*N*=7402; missing=515)							
Never	1048	0.155	0.362	0.129	0.335	−0.026	0.001
Monthly or less	1610	0.215	0.411	0.219	0.414	0.004	0.663
2–4 times a month	1620	0.206	0.404	0.231	0.421	0.025	0.010
2–3 times a week	2104	0.277	0.448	0.291	0.454	0.014	0.180
4 times or more a week	1020	0.146	0.354	0.130	0.336	−0.016	0.041
Using drugs (*N*=7374; missing=543)							
Yes	158	0.029	0.168	0.014	0.119	−0.015	0.000
No	7216	0.971	0.168	0.986	0.119	0.015	0.000
Experienced suicidal thoughts (*N*=7360; missing=557)							
Yes	2309	0.320	0.467	0.308	0.462	−0.012	0.264
No	5051	0.680	0.467	0.692	0.462	0.012	0.264
Having mental health support from (*N*=7917; missing=0)^a^							
No support currently	6571	0.825	0.380	0.835	0.371	0.010	0.216
GP	1041	0.127	0.333	0.136	0.343	0.009	0.235
Counsellor/Psychologist/Psychotherapist	359	0.052	0.223	0.038	0.192	−0.014	0.002
Community Mental Health Team	136	0.026	0.158	0.009	0.093	−0.017	0.000
Inpatient in a psychiatric hospital	0						
Religious/Spiritual Leader	43	0.005	0.071	0.006	0.076	0.001	0.616
Other	156	0.022	0.148	0.017	0.129	−0.005	0.090

*Note:* Mean is a proportion of individual in a category. If we multiply means by 100, we will get percentages. SD is standard deviation.

a Because of multiple responses from some individuals, total responses are 8306, which is higher than our total respondents, 7917. For example, one individual might have taken mental health support from both GP and community mental health team.

### 9.4 Part C: COVID-19 information and advice

The most common sources of information about coronavirus reported are TV news programmes (18.30%), social media (10.37%), Government briefings (13.70%), News apps (9.75%), the NHS website (10.40%), Gov.uk website (9.67%), and employer (15.07%). Participants are most likely to find that social media stories make them feel worried and fearful but they are unlikely to believe the information with only about 10% noting they believe social media stories. There are multiple sources of data that are not mutually exclusive for the participants. Table 3 shows the summary statistics of attitudes and health outcomes due to coronavirus analysed by HCP. This table indicates that HCP are more compliant with government advice (several times and most of the time) and in higher proportions than others, with rare engagement in risky activities (e.g., going shops, party, and social gathering frequently) being significantly lower than the counterparts.

**Table 3:** Summary statistics of attitudes and health outcomes in coronavirus time by healthcare professional.

		Non-healthcare Professional (*N* = 3984)		Healthcare Professional (*N* = 3933)		Mean Diff.	p-value
	*N*	Mean	SD	Mean	SD		
Followed government advice (*N*=7917; missing=0)							
Very few time	683	0.140	0.347	0.032	0.177	−0.107	0.000
Some of the time	135	0.016	0.124	0.019	0.135	0.003	0.303
Several time	1336	0.153	0.360	0.185	0.388	0.032	0.000
Most of the time	5763	0.692	0.462	0.764	0.424	0.072	0.000
Did risky activities (*N*=7917; missing=0)							
Rare	7587	0.964	0.186	0.952	0.213	−0.012	0.009
Very few time	312	0.034	0.181	0.045	0.207	0.011	0.011
Some of the time	8	0.001	0.027	0.001	0.036	0.001	0.468
Several time	2	0.000	0.016	0.000	0.016	0.000	0.993
Most of the time	8	0.001	0.032	0.001	0.032	0.000	0.985
Patient health questionnaire (PHQ-9) (*N*=7917; missing=0)							
None (0–4)	3501	0.463	0.499	0.421	0.494	−0.042	0.000
Mild (5–9)	2202	0.255	0.436	0.302	0.459	0.047	0.000
Moderate (10–14)	1146	0.138	0.345	0.152	0.359	0.014	0.077
Moderately Severe (15–19)	618	0.074	0.262	0.082	0.275	0.008	0.180
Severe (20–27)	450	0.070	0.256	0.043	0.203	−0.027	0.000
Generalised anxiety disorder (GAD-7) (*N*=7917; missing=0)							
None (0–4)	4545	0.580	0.494	0.569	0.495	−0.011	0.320
Mild (5–9)	1942	0.235	0.424	0.256	0.436	0.020	0.035
Moderate (10–14)	816	0.100	0.301	0.106	0.308	0.005	0.432
Severe (15–21)	614	0.085	0.279	0.070	0.255	−0.015	0.015
Impact of events scale-revised (IES-R) (*N*=7917; missing=0)							
None (0–23)	5892	0.741	0.438	0.747	0.435	0.006	0.572
PTSD may be concern (24–32)	757	0.095	0.293	0.096	0.295	0.001	0.882
Probably PTSD diagnosis (33–38)	352	0.042	0.202	0.047	0.211	0.004	0.375
High PTSD (39 and above)	916	0.121	0.326	0.110	0.313	−0.011	0.139
Drinking alcohol changed (*N*=5459; missing=2458)							
Decreased	725	0.130	0.336	0.136	0.343	0.006	0.497
Unchanged	2925	0.557	0.497	0.517	0.500	−0.041	0.003
Increased	1809	0.313	0.464	0.348	0.476	0.034	0.007
Drug use changed (*N*=152; missing=7765)							
Decreased	41	0.286	0.454	0.241	0.432	−0.045	0.553
Unchanged	64	0.439	0.499	0.389	0.492	−0.050	0.554
Increased	47	0.276	0.449	0.370	0.487	0.095	0.229
Mental health support changed (*N*=1396; missing=6521)							
Decreased	260	0.203	0.403	0.168	0.374	−0.035	0.090
Unchanged	970	0.694	0.461	0.695	0.461	0.001	0.966
Increased	166	0.102	0.303	0.137	0.344	0.034	0.048
Mental health affected (*N*=2629; missing=5288)							
No	729	0.264	0.441	0.291	0.454	0.026	0.129
Some of the time	1118	0.418	0.493	0.433	0.496	0.014	0.455
Most of the time	395	0.152	0.360	0.148	0.355	−0.004	0.750
All of the time	387	0.165	0.372	0.129	0.335	−0.036	0.008
Mental health changed (*N*=2611; missing=5306)							
Decreased	1479	0.565	0.496	0.567	0.496	0.002	0.917
Unchanged	928	0.351	0.477	0.360	0.480	0.009	0.623
Increased	204	0.084	0.277	0.072	0.259	−0.011	0.285
Suicidal thoughts changed (*N*=232; missing=7685)							
Yes	188	0.815	0.390	0.802	0.401	−0.012	0.824
No	44	0.185	0.390	0.198	0.401	0.012	0.824
Worried about corona virus (*N*=7917; missing=0)							
Not at all	773	0.151	0.358	0.044	0.205	−0.107	0.000
A little bit	859	0.098	0.298	0.119	0.324	0.020	0.004
Moderately	2596	0.294	0.456	0.363	0.481	0.069	0.000
Quite a bit	2466	0.302	0.459	0.321	0.467	0.018	0.081
Extremely	1223	0.155	0.362	0.154	0.361	−0.001	0.923

*Note:* Mean is a proportion of individual in a category. If we multiply means by 100, we will get percentages. SD is standard deviation.

### 9.5 Part D: psychological impact

Findings in Table 3 imply that HCP have mild depression and anxiety in higher proportions than others.

Alcohol intake increased in significantly higher proportions among HCP than others, but drug use remains the same in both groups. The increase in mental health support is higher among HCP than others. Non-HCP have more worries than HCP, as mean differences are significantly higher in the categories a little bit and moderately.

### 9.6 Part E: coping strategies

Table 4 shows the summary statistics of coping mechanisms by non-HCP and HCP. The analysis shows that daily communication with friends and family is significantly higher among HCP than others. In spite of this, they report as feeling more isolated from friends and family than non-HCP.

**Table 4:** Summary statistics of coping mechanisms in coronavirus time by healthcare professional.

		Non-healthcare Professional (*N* = 3984)		Healthcare Professional (*N* = 3933)		Mean Diff.	p-value
	*N*	Mean	SD	Mean	SD		
Communicated with friends/family (*N*=7917; missing=0)							
Not at all	1026	0.182	0.386	0.076	0.265	−0.106	0.000
Every few days	1595	0.200	0.400	0.203	0.403	0.004	0.669
Daily	4057	0.471	0.499	0.555	0.497	0.084	0.000
Several times a day	1239	0.147	0.354	0.166	0.372	0.018	0.024
Relationships impacted (*N*=6796; missing=1121)							
Isolated	2912	0.408	0.492	0.446	0.497	0.038	0.002
No change	1300	0.205	0.404	0.179	0.384	−0.026	0.007
Feeling closer	1177	0.177	0.381	0.170	0.376	−0.007	0.473
Having more arguments	209	0.032	0.175	0.030	0.171	−0.002	0.690
Talking more	1198	0.179	0.383	0.174	0.379	−0.004	0.641
Did good/coping activities (*N*=7917; missing=0)							
Not at all	1007	0.180	0.384	0.073	0.261	−0.107	0.000
Every few days	2811	0.339	0.474	0.371	0.483	0.032	0.003
Daily	4051	0.473	0.499	0.550	0.498	0.077	0.000
Several times a day	48	0.007	0.084	0.005	0.071	−0.002	0.266
Good/coping activities changed (*N*=7917; missing=0)							
Decreased	790	0.097	0.295	0.103	0.304	0.006	0.347
Unchanged	1706	0.260	0.439	0.170	0.376	−0.090	0.000
Increased	5421	0.643	0.479	0.727	0.446	0.083	0.000
Amount of activity time impacted (*N*=6794; missing=1123)							
None at all	327	0.046	0.210	0.050	0.217	0.003	0.512
A little	727	0.105	0.307	0.109	0.311	0.003	0.644
A moderate amount	1724	0.248	0.432	0.259	0.438	0.010	0.322
A lot	1695	0.249	0.433	0.250	0.433	0.001	0.951
A great deal	2321	0.351	0.477	0.333	0.471	−0.018	0.118
Confident on coping (*N*=6851; missing=1066)							
Not at all	50	0.012	0.109	0.003	0.055	−0.009	0.000
A little bit	454	0.080	0.271	0.054	0.227	−0.025	0.000
Moderately	1668	0.267	0.442	0.223	0.416	−0.044	0.000
Quite a bit	2952	0.414	0.493	0.446	0.497	0.033	0.006
Extremely	1727	0.228	0.420	0.274	0.446	0.046	0.000

*Note:* Mean is a proportion of individual in a category. If we multiply means by 100, we will get percentages. SD is standard deviation.

HCP show higher coping activities compared to others. The impact on coping activity time is the same between the two groups. However, HCP report significantly higher confidence in coping than others.

### 9.7 Ordered logit regressions

Table 5, Table 6, Table 7, Table 8 show results of ordered logit regressions of all outcomes listed in Tables 3 and 4. Coefficients in Table 5, Table 6, Table 7, Table 8 are changes in log-odds ratios due to changes in covariates (demographics and pre-existing health conditions and wellbeing). The signs of coefficients imply in which direction covariates affect outcomes.

**Table 5:** Ordered logit regressions of ordered psychological health outcomes on healthcare professionals and other covariates.

	Government advice		Risky activities		PHQ-9		GAD-7		IESR	
	Coeff.	p-value	Coeff.	p-value	Coeff.	p-value	Coeff.	p-value	Coeff.	p-value
Healthcare Professionals	0.018	(0.841)	0.085	(0.653)	−0.111	(0.114)	−0.180⁎⁎	(0.016)	−0.064	(0.467)
Age Category (Base: below 21)										
21–24	0.348	(0.388)	−0.214	(0.751)	0.268	(0.538)	0.137	(0.674)	0.226	(0.495)
25–34	0.430	(0.271)	−0.536	(0.413)	−0.196	(0.651)	−0.153	(0.627)	−0.122	(0.706)
35–44	0.766*	(0.053)	−1.005	(0.126)	−0.286	(0.513)	−0.202	(0.524)	−0.020	(0.950)
45–54	0.911⁎⁎	(0.021)	−0.828	(0.206)	−0.699	(0.111)	−0.636⁎⁎	(0.046)	−0.313	(0.341)
55–64	0.952⁎⁎	(0.017)	−1.123*	(0.093)	−0.846*	(0.054)	−0.791⁎⁎	(0.014)	−0.344	(0.300)
65 and over	1.252⁎⁎	(0.012)	−1.128	(0.200)	−1.139⁎⁎	(0.019)	−1.053⁎⁎⁎	(0.008)	−0.563	(0.195)
Ethnicity (Base: others)										
White	0.085	(0.567)	0.328	(0.328)	0.082	(0.530)	0.134	(0.309)	0.161	(0.295)
Asian	0.091	(0.749)	0.631	(0.244)	−0.256	(0.286)	−0.006	(0.980)	−0.074	(0.818)
Black	−0.218	(0.524)	0.474	(0.512)	−0.532	(0.119)	−0.143	(0.645)	−0.348	(0.392)
Religion (Base: others)										
Christian	−0.013	(0.953)	−0.513	(0.183)	0.282	(0.123)	0.336*	(0.067)	0.232	(0.285)
Muslim	−0.593	(0.166)	−0.504	(0.615)	−0.175	(0.631)	−0.132	(0.753)	0.043	(0.929)
Sikh	0.038	(0.955)	−0.184	(0.873)	−0.824	(0.109)	−0.457	(0.487)	−0.816	(0.505)
Hindu	−0.707	(0.106)	−0.566	(0.486)	0.262	(0.492)	0.454	(0.200)	0.599	(0.205)
Jewish	−0.607	(0.223)	−13.924⁎⁎⁎	(0.000)	−0.128	(0.802)	0.020	(0.965)	0.108	(0.839)
Buddhist	0.166	(0.694)	0.048	(0.938)	0.059	(0.885)	0.141	(0.718)	0.191	(0.684)
No religion	−0.050	(0.817)	−0.341	(0.368)	0.260	(0.154)	0.161	(0.381)	0.153	(0.481)
Male	−0.540⁎⁎⁎	(0.000)	0.278	(0.101)	−0.421⁎⁎⁎	(0.000)	−0.419⁎⁎⁎	(0.000)	−0.677⁎⁎⁎	(0.000)
Left education (Base: before age 16)										
At age 16	0.007	(0.967)	0.156	(0.624)	0.160	(0.200)	0.016	(0.902)	0.078	(0.599)
At age 18	−0.103	(0.524)	−0.178	(0.579)	0.229⁎⁎	(0.048)	0.086	(0.472)	0.045	(0.751)
Attended University	−0.263*	(0.072)	0.064	(0.814)	−0.107	(0.303)	−0.202*	(0.063)	−0.171	(0.184)
Accommodation (Base: others)										
Own home	0.388*	(0.064)	−0.272	(0.429)	−0.566⁎⁎⁎	(0.002)	−0.610⁎⁎⁎	(0.002)	−0.533⁎⁎⁎	(0.004)
Shared	0.580*	(0.065)	−1.221*	(0.063)	−0.350	(0.195)	−0.790⁎⁎⁎	(0.006)	−0.307	(0.312)
Private rented	0.353	(0.107)	−0.382	(0.294)	−0.303	(0.107)	−0.369*	(0.069)	−0.299	(0.128)
Parent's home										
Own home	0.318	(0.188)	−0.675	(0.104)	−0.177	(0.400)	−0.331	(0.135)	−0.285	(0.200)
Keyworker (Base: no)										
Health and Social	−0.201*	(0.061)	0.060	(0.785)	−0.015	(0.850)	0.034	(0.687)	−0.064	(0.522)
Education	−0.036	(0.839)	0.030	(0.930)	−0.021	(0.860)	0.102	(0.450)	0.131	(0.378)
Key public services	−0.839⁎⁎⁎	(0.005)	0.781	(0.122)	0.049	(0.821)	−0.144	(0.524)	−0.220	(0.522)
Local government	0.236	(0.399)	0.096	(0.841)	0.076	(0.653)	−0.004	(0.983)	0.231	(0.280)
Food	−0.788⁎⁎⁎	(0.005)	0.807*	(0.058)	0.579⁎⁎⁎	(0.006)	0.408*	(0.059)	0.242	(0.318)
Public safety	−0.360	(0.499)	0.445	(0.685)	0.076	(0.899)	0.091	(0.844)	−0.891	(0.247)
Transport	0.889	(0.169)	−13.793⁎⁎⁎	(0.000)	0.248	(0.549)	0.272	(0.541)	0.501	(0.147)
Utilities	−0.176	(0.543)	0.138	(0.804)	0.088	(0.696)	0.048	(0.823)	0.303	(0.216)
Pre-existing health condition (Base: none)										
Anxiety	0.061	(0.656)	−0.389	(0.174)	0.358⁎⁎⁎	(0.000)	0.726⁎⁎⁎	(0.000)	0.585⁎⁎⁎	(0.000)
Panic Attacks	−0.351	(0.162)	−0.010	(0.984)	0.623⁎⁎⁎	(0.000)	1.214⁎⁎⁎	(0.000)	1.182⁎⁎⁎	(0.000)
Anorexia	−0.308	(0.642)	−13.942⁎⁎⁎	(0.000)	0.565	(0.314)	0.469	(0.479)	0.518	(0.498)
Psychosis	−0.566	(0.652)	−13.416⁎⁎⁎	(0.000)	−0.547	(0.589)	−14.335⁎⁎⁎	(0.000)	0.508	(0.786)
Depression	−0.050	(0.646)	−0.162	(0.462)	0.834⁎⁎⁎	(0.000)	0.757⁎⁎⁎	(0.000)	0.617⁎⁎⁎	(0.000)
Bulimia	−0.694*	(0.097)	−0.328	(0.764)	0.818*	(0.057)	1.081⁎⁎⁎	(0.003)	0.111	(0.841)
Social phobia	−0.477	(0.154)	0.297	(0.595)	1.200⁎⁎⁎	(0.000)	1.391⁎⁎⁎	(0.000)	1.563⁎⁎⁎	(0.000)
Attention deficit	−0.747	(0.180)	−0.530	(0.645)	1.049⁎⁎	(0.013)	0.918⁎⁎	(0.023)	0.934*	(0.050)
Obsessive	0.213	(0.414)	−0.650	(0.301)	0.747⁎⁎⁎	(0.002)	1.127⁎⁎⁎	(0.000)	0.860⁎⁎⁎	(0.000)
Post-Traumatic	0.293	(0.188)	−0.481	(0.241)	0.956⁎⁎⁎	(0.000)	1.174⁎⁎⁎	(0.000)	1.281⁎⁎⁎	(0.000)
Alcohol/Drug	0.148	(0.887)	0.247	(0.831)	0.967*	(0.063)	1.229⁎⁎	(0.029)	1.719⁎⁎⁎	(0.000)
Bi-polar disorder	−0.945⁎⁎	(0.016)	0.776	(0.134)	0.471	(0.208)	0.117	(0.751)	0.372	(0.380)
Personality disorder	−0.627⁎⁎	(0.028)	0.016	(0.976)	1.343⁎⁎⁎	(0.000)	1.193⁎⁎⁎	(0.000)	1.194⁎⁎⁎	(0.000)
Drinking alcohol (Base: Never)										
Monthly or less	0.009	(0.939)	0.141	(0.523)	0.019	(0.835)	0.039	(0.688)	−0.104	(0.357)
2–4 times a month	−0.153	(0.198)	−0.133	(0.561)	−0.101	(0.275)	−0.068	(0.476)	−0.173	(0.120)
2–3 times a week	−0.088	(0.445)	−0.211	(0.349)	−0.006	(0.946)	0.011	(0.901)	−0.002	(0.986)
More a week	−0.277⁎⁎	(0.038)	0.229	(0.351)	0.224⁎⁎	(0.030)	0.241⁎⁎	(0.023)	0.350⁎⁎⁎	(0.004)
Using drugs	−0.515⁎⁎	(0.015)	0.810⁎⁎	(0.016)	0.336*	(0.096)	0.376*	(0.063)	−0.018	(0.938)
Suicidal thoughts	−0.261⁎⁎⁎	(0.001)	0.409⁎⁎⁎	(0.007)	0.891⁎⁎⁎	(0.000)	0.592⁎⁎⁎	(0.000)	0.749⁎⁎⁎	(0.000)
Having mental health support from (Base: No)										
GP	0.146	(0.202)	0.326	(0.113)	0.363⁎⁎⁎	(0.000)	0.199⁎⁎	(0.028)	0.189⁎⁎	(0.047)
Counsellor etc.	−0.216	(0.169)	0.271	(0.315)	0.414⁎⁎⁎	(0.001)	0.446⁎⁎⁎	(0.002)	0.361⁎⁎⁎	(0.010)
Community Mental	0.074	(0.793)	−0.601	(0.227)	0.506*	(0.070)	0.526*	(0.059)	0.196	(0.446)
Psychiatric hospital										
Religious Leader	0.013	(0.975)	−0.800	(0.446)	−0.600⁎⁎	(0.042)	−0.673*	(0.073)	−0.812*	(0.078)
Constant1	−3.723⁎⁎⁎	(0.000)	2.146⁎⁎	(0.012)	−0.722	(0.163)	−0.160	(0.713)	1.041⁎⁎	(0.020)
Constant2	−2.961⁎⁎⁎	(0.000)	5.079⁎⁎⁎	(0.000)	0.819	(0.114)	1.334⁎⁎⁎	(0.002)	1.735⁎⁎⁎	(0.000)
Constant3	−0.775	(0.139)	5.709⁎⁎⁎	(0.000)	1.997⁎⁎⁎	(0.000)	2.424⁎⁎⁎	(0.000)	2.202⁎⁎⁎	(0.000)
Constant4			5.997⁎⁎⁎	(0.000)	3.194⁎⁎⁎	(0.000)				
Observations	5655		5655		5655		5655		5655	
Adjusted *R*^2^	0.029		0.035		0.087		0.077		0.076	

*Note: p*-values are in parentheses.

⁎ *p* < 0.10.

⁎⁎ *p* < 0.05.

⁎⁎⁎ *p* < 0.01.

**Table 6:** Ordered logit regressions of ordered health change outcomes on healthcare professionals and other demographics.

	Drinking alcohol changed		drug use changed		Mental health support changed		Mental health affected		Mental health changed	
	Coeff.	p-value	Coeff.	p-value	Coeff.	p-value	Coeff.	p-value	Coeff.	p-value
Healthcare Professionals	0.056	(0.511)	0.666	(0.446)	0.126	(0.532)	0.087	(0.462)	−0.067	(0.584)
Age Category (Base: below 21)										
21–24	0.317	(0.460)	20.362⁎⁎⁎	(0.000)	0.285	(0.841)	0.382	(0.306)	0.407	(0.508)
25–34	0.644	(0.117)	20.435⁎⁎⁎	(0.000)	0.345	(0.807)	−0.139	(0.690)	0.618	(0.303)
35–44	0.528	(0.205)	20.196⁎⁎⁎	(0.000)	0.287	(0.840)	−0.275	(0.437)	0.655	(0.277)
45–54	0.122	(0.769)	19.381⁎⁎⁎	(0.000)	0.064	(0.964)	−0.511	(0.153)	1.046*	(0.083)
55–64	−0.112	(0.788)	19.373⁎⁎⁎	(0.000)	0.194	(0.891)	−0.657*	(0.067)	1.296⁎⁎	(0.033)
65 and over	0.004	(0.993)	0.000	(.)	−0.559	(0.702)	−0.532	(0.285)	1.250*	(0.059)
Ethnicity (Base: others)										
White	−0.026	(0.855)	−1.184	(0.205)	−0.684*	(0.075)	−0.208	(0.335)	−0.176	(0.431)
Asian	−0.365	(0.192)	−2.936	(0.691)	−2.459⁎⁎⁎	(0.001)	0.522	(0.281)	−0.052	(0.920)
Black	0.232	(0.438)	−37.276⁎⁎⁎	(0.000)	−2.431⁎⁎	(0.029)	0.010	(0.990)	−0.491	(0.446)
Religion (Base: others)										
Christian	0.220	(0.317)	0.908	(0.521)	−0.328	(0.449)	0.335	(0.167)	−0.843⁎⁎⁎	(0.004)
Muslim	0.179	(0.603)	27.270⁎⁎⁎	(0.000)	−1.245	(0.185)	−0.224	(0.668)	0.127	(0.828)
Sikh	−0.681	(0.463)	0.000	(.)	0.000	(.)	−15.683⁎⁎⁎	(0.000)	1.831	(0.267)
Hindu	−0.039	(0.931)	21.338⁎⁎⁎	(0.004)	1.221	(0.135)	−0.357	(0.579)	−0.506	(0.454)
Jewish	−0.117	(0.749)	0.000	(.)	1.645*	(0.099)	0.173	(0.802)	−2.209⁎⁎	(0.011)
Buddhist	−0.226	(0.685)	−0.578	(0.781)	−0.920	(0.360)	1.569⁎⁎	(0.015)	−1.036	(0.146)
No religion	0.108	(0.624)	1.888	(0.186)	−0.289	(0.500)	0.176	(0.467)	−0.745⁎⁎	(0.012)
Male	−0.386⁎⁎⁎	(0.000)	−1.059	(0.165)	−0.007	(0.977)	−0.290⁎⁎	(0.047)	0.043	(0.765)
Left education (Base: before age 16)										
At age 16	0.194	(0.167)	−0.700	(0.683)	−0.274	(0.446)	0.118	(0.550)	−0.163	(0.441)
At age 18	0.275⁎⁎	(0.043)	0.690	(0.707)	−0.278	(0.455)	0.212	(0.259)	−0.151	(0.466)
Attended University	0.122	(0.312)	−0.802	(0.627)	−0.358	(0.304)	−0.055	(0.758)	0.055	(0.776)
Accommodation (Base: others)										
Own home	0.179	(0.310)	1.198	(0.393)	0.158	(0.698)	−0.516⁎⁎	(0.021)	0.540*	(0.055)
Shared	0.031	(0.917)	−1.799	(0.132)	0.188	(0.819)	−0.585*	(0.063)	0.331	(0.464)
Private rented	0.233	(0.212)	−0.001	(0.999)	0.186	(0.668)	−0.491⁎⁎	(0.035)	0.232	(0.432)
Parent's home	0.000	(.)	0.000	(.)	0.000	(.)	0.000	(.)	0.000	(.)
Own home	−0.460⁎⁎	(0.048)	−1.694	(0.142)	0.324	(0.526)	−0.788⁎⁎⁎	(0.004)	0.340	(0.319)
Keyworker (Base: no)										
Health and Social	−0.041	(0.688)	−0.169	(0.853)	0.042	(0.861)	−0.153	(0.261)	−0.057	(0.688)
Education	0.147	(0.356)	1.758*	(0.072)	−0.301	(0.378)	−0.230	(0.245)	−0.117	(0.646)
Key public services	−0.007	(0.981)	0.000	(.)	−0.201	(0.795)	−0.001	(0.999)	0.268	(0.558)
Local government	0.111	(0.632)	17.361⁎⁎⁎	(0.000)	0.159	(0.793)	−0.390	(0.226)	0.429	(0.149)
Food	−0.097	(0.730)	2.176⁎⁎	(0.014)	−0.824	(0.118)	0.517	(0.173)	−0.625	(0.122)
Public safety	0.283	(0.533)	0.000	(.)	0.503	(0.396)	−1.019	(0.121)	0.847*	(0.050)
Transport	0.987*	(0.062)	−1.401	(0.400)	0.836	(0.732)	0.404	(0.442)	0.195	(0.857)
Utilities	0.562*	(0.075)	−41.473⁎⁎⁎	(0.000)	1.059	(0.166)	0.264	(0.398)	−0.214	(0.584)
Pre-existing health condition (Base: none)										
Anxiety	0.114	(0.358)	−1.301	(0.392)	0.838*	(0.067)	−0.188	(0.347)	0.306	(0.117)
Panic Attacks	0.322	(0.280)	−2.688⁎⁎	(0.038)	0.558	(0.317)	0.287	(0.304)	−0.114	(0.658)
Anorexia	0.851⁎⁎	(0.011)	0.000	(.)	0.580	(0.311)	−0.210	(0.788)	0.576	(0.389)
Psychosis	−1.865⁎⁎⁎	(0.000)	0.000	(.)	−14.658⁎⁎⁎	(0.000)	−1.826	(0.168)	2.679⁎⁎	(0.011)
Depression	0.064	(0.557)	−0.053	(0.962)	0.580	(0.188)	−0.095	(0.618)	0.182	(0.329)
Bulimia	−0.049	(0.872)	−1.074	(0.727)	−0.124	(0.875)	−0.079	(0.876)	−0.243	(0.658)
Social phobia	0.858⁎⁎	(0.019)	0.038	(0.985)	0.281	(0.675)	0.462	(0.206)	0.092	(0.797)
Attention deficit	1.671⁎⁎⁎	(0.001)	−2.231	(0.243)	0.104	(0.904)	0.465	(0.347)	−0.156	(0.772)
Obsessive	0.237	(0.356)	0.527	(0.761)	0.675	(0.245)	0.312	(0.264)	−0.062	(0.833)
Post-Traumatic	0.256	(0.148)	−1.775	(0.284)	−0.172	(0.744)	0.319	(0.176)	0.045	(0.854)
Alcohol/Drug	0.029	(0.971)	−0.502	(0.638)	−0.900	(0.398)	1.203	(0.109)	−0.437	(0.573)
Bi-polar disorder	0.030	(0.935)	53.481⁎⁎⁎	(0.000)	0.335	(0.643)	−0.505	(0.212)	1.457⁎⁎⁎	(0.002)
Personality disorder	0.647⁎⁎	(0.045)	15.750⁎⁎	(0.023)	0.597	(0.337)	0.782⁎⁎	(0.020)	−0.428	(0.298)
Drinking alcohol (Base: Never)										
Monthly or less	−0.444⁎⁎⁎	(0.000)	0.101	(0.914)	−0.131	(0.569)	0.098	(0.490)	0.168	(0.250)
2–4 times a month	0.430⁎⁎⁎	(0.000)	−0.796	(0.473)	0.045	(0.852)	−0.048	(0.733)	0.097	(0.524)
2–3 times a week	1.708⁎⁎⁎	(0.000)	−1.256	(0.157)	−0.016	(0.946)	−0.010	(0.942)	0.197	(0.183)
More a week	2.651⁎⁎⁎	(0.000)	−0.785	(0.493)	0.034	(0.898)	0.313*	(0.061)	−0.158	(0.368)
Using drugs	−0.048	(0.870)	0.000	(.)	−0.155	(0.711)	0.156	(0.578)	0.074	(0.825)
Suicidal thoughts	0.052	(0.514)	1.338*	(0.081)	−0.292*	(0.055)	0.087	(0.323)	−0.250⁎⁎⁎	(0.010)
Having mental health support from (Base: No)										
GP	0.101	(0.361)	1.044	(0.249)	−0.387	(0.129)	0.181⁎⁎	(0.044)	−0.227⁎⁎	(0.018)
Counsellor etc.	−0.306*	(0.051)	0.396	(0.662)	0.191	(0.454)	0.740⁎⁎⁎	(0.000)	−0.060	(0.704)
Community Mental	−0.219	(0.427)	17.243⁎⁎⁎	(0.000)	−0.781*	(0.066)	0.907⁎⁎⁎	(0.000)	−0.530*	(0.096)
Psychiatric hospital	0.000	(.)	0.000	(.)	0.000	(.)	0.000	(.)	0.000	(.)
Religious Leader	0.447	(0.243)	−35.565⁎⁎⁎	(0.000)	0.284	(0.420)	0.161	(0.645)	−0.365	(0.384)
Constant1	−0.732	(0.159)	18.655⁎⁎⁎	(0.000)	−2.590	(0.118)	−1.564⁎⁎⁎	(0.005)	0.489	(0.520)
Constant2	2.375⁎⁎⁎	(0.000)	21.836⁎⁎⁎	(0.000)	1.328	(0.422)	0.481	(0.392)	2.818⁎⁎⁎	(0.000)
Constant3							1.466⁎⁎⁎	(0.009)		
Observations	4238		104		1039		2024		2013	
Adjusted *R*^2^	0.140		0.346		0.051		0.042		0.042	

*Note: p*-values are in parentheses.

⁎ *p* < 0.10.

⁎⁎ *p* < 0.05.

⁎⁎⁎ *p* < 0.01.

**Table 7:** Ordered logit regressions of ordered health change outcomes on healthcare professionals and other covariates.

	Suicidal thoughts changed		Worried about corona virus		Communication with family		Relationships impacted		Did good/coping activities	
	Coeff.	p-value	Coeff.	p-value	Coeff.	p-value	Coeff.	p-value	Coeff.	p-value
Healthcare Professionals	−0.322	(0.657)	−0.118*	(0.083)	0.092	(0.186)	−0.046	(0.507)	0.078	(0.291)
Age Category (Base: below 21)										
21–24	−16.946	(.)	0.342	(0.290)	−0.226	(0.501)	−0.010	(0.979)	0.245	(0.412)
25–34	−18.254	(.)	0.355	(0.260)	−0.386	(0.240)	0.080	(0.835)	0.206	(0.486)
35–44	−19.225	(.)	0.411	(0.197)	−0.695⁎⁎	(0.037)	0.050	(0.898)	0.502*	(0.095)
45–54	−18.624	(.)	0.286	(0.370)	−0.915⁎⁎⁎	(0.006)	0.100	(0.797)	0.151	(0.617)
55–64	−18.313	(.)	0.339	(0.291)	−0.995⁎⁎⁎	(0.003)	0.122	(0.755)	0.107	(0.726)
65 and over	−1.858	(.)	0.139	(0.731)	−1.470⁎⁎⁎	(0.000)	0.012	(0.977)	0.460	(0.205)
Ethnicity (Base: others)										
White	−0.511	(0.693)	−0.006	(0.960)	0.111	(0.353)	−0.324⁎⁎⁎	(0.003)	0.061	(0.615)
Asian	18.452⁎⁎⁎	(0.000)	0.099	(0.681)	−0.073	(0.776)	0.437⁎⁎	(0.043)	0.297	(0.253)
Black	−1.233	(0.743)	0.187	(0.631)	0.134	(0.736)	−0.054	(0.823)	−0.278	(0.427)
Religion (Base: others)										
Christian	−17.903	(.)	0.440⁎⁎	(0.019)	0.310*	(0.062)	0.180	(0.309)	0.322*	(0.071)
Muslim	−0.000	(.)	0.100	(0.803)	0.834⁎⁎	(0.019)	−0.470	(0.138)	1.163⁎⁎	(0.012)
Sikh	−0.000	(.)	0.489	(0.355)	2.304⁎⁎⁎	(0.005)	−0.112	(0.796)	1.438*	(0.084)
Hindu	−17.404	(.)	0.013	(0.973)	0.366	(0.369)	−0.207	(0.569)	0.063	(0.883)
Jewish	1.374	(.)	−0.452	(0.354)	0.642	(0.147)	0.317	(0.389)	0.352	(0.443)
Buddhist	−20.294⁎⁎⁎	(0.001)	0.221	(0.520)	0.191	(0.565)	−0.139	(0.636)	0.603	(0.125)
No religion	−18.037	(.)	0.151	(0.417)	0.056	(0.731)	0.162	(0.361)	0.037	(0.835)
Male	−0.281	(0.775)	−0.281⁎⁎⁎	(0.000)	−0.527⁎⁎⁎	(0.000)	0.111*	(0.099)	−0.096	(0.225)
Left education (Base: before age 16)										
At age 16	−0.150	(0.926)	0.212	(0.101)	−0.010	(0.939)	0.101	(0.396)	−0.203	(0.104)
At age 18	0.139	(0.938)	0.070	(0.567)	−0.043	(0.717)	0.075	(0.515)	−0.166	(0.166)
Attended University	0.167	(0.907)	−0.239⁎⁎	(0.028)	0.053	(0.596)	0.284⁎⁎⁎	(0.005)	0.047	(0.654)
Accommodation (Base: others)										
Own home	−1.337	(0.314)	−0.271	(0.177)	0.222	(0.133)	0.132	(0.405)	0.464⁎⁎⁎	(0.003)
Shared	−2.877*	(0.086)	−0.414	(0.116)	−0.214	(0.373)	0.112	(0.663)	−0.692⁎⁎⁎	(0.004)
Private rented	−1.122	(0.486)	−0.159	(0.442)	0.317⁎⁎	(0.045)	−0.041	(0.808)	0.043	(0.798)
Parent's home	−0.000	(.)	0.000	(.)	0.000	(.)	0.000	(.)	0.000	(.)
Own home	−2.689*	(0.082)	0.100	(0.645)	0.245	(0.195)	0.282	(0.147)	−0.149	(0.436)
Keyworker (Base: no)										
Health and Social	−0.002	(0.998)	−0.109	(0.159)	−0.233⁎⁎⁎	(0.004)	−0.188⁎⁎	(0.019)	−0.135	(0.115)
Education	1.228	(0.419)	−0.184	(0.128)	0.340⁎⁎⁎	(0.009)	0.107	(0.423)	0.410⁎⁎⁎	(0.005)
Key public services	−2.395	(0.149)	−0.526*	(0.055)	−0.037	(0.887)	−0.190	(0.509)	0.327	(0.247)
Local government	19.234⁎⁎⁎	(0.000)	−0.204	(0.289)	−0.047	(0.804)	−0.157	(0.402)	−0.095	(0.622)
Food	18.211⁎⁎⁎	(0.000)	−0.146	(0.523)	−0.002	(0.994)	−0.120	(0.566)	−0.210	(0.383)
Public safety	−0.000	(.)	−0.648*	(0.092)	0.116	(0.762)	−0.140	(0.719)	0.536	(0.426)
Transport	18.945⁎⁎⁎	(0.000)	0.187	(0.571)	−0.290	(0.416)	0.161	(0.615)	−0.230	(0.514)
Utilities	20.100⁎⁎⁎	(0.000)	−0.342	(0.203)	0.199	(0.416)	−0.111	(0.630)	−0.318	(0.164)
Pre-existing health condition (Base: none)										
Anxiety	19.058⁎⁎⁎	(0.000)	0.197⁎⁎	(0.040)	0.138	(0.196)	−0.098	(0.400)	0.022	(0.841)
Panic Attacks	18.453⁎⁎⁎	(0.000)	0.846⁎⁎⁎	(0.000)	0.643⁎⁎	(0.010)	−0.008	(0.971)	0.001	(0.995)
Anorexia	−0.000	(.)	0.165	(0.774)	0.147	(0.855)	0.372	(0.450)	−0.261	(0.655)
Psychosis	−0.000	(.)	−1.573*	(0.056)	0.636	(0.704)	0.155	(0.820)	0.622	(0.470)
Depression	0.722	(0.453)	0.309⁎⁎⁎	(0.000)	−0.046	(0.593)	−0.089	(0.329)	−0.036	(0.686)
Bulimia	18.710⁎⁎⁎	(0.000)	0.350	(0.381)	0.031	(0.938)	0.308	(0.440)	−0.237	(0.516)
Social phobia	−1.018	(0.443)	0.451*	(0.096)	−0.461*	(0.055)	0.279	(0.297)	−0.227	(0.388)
Attention deficit	17.010⁎⁎⁎	(0.000)	0.487	(0.179)	−0.201	(0.676)	−0.645*	(0.093)	−0.032	(0.944)
Obsessive	1.573	(0.362)	0.835⁎⁎⁎	(0.000)	−0.042	(0.829)	−0.092	(0.684)	0.013	(0.955)
Post-Traumatic	0.574	(0.510)	0.206	(0.183)	−0.021	(0.901)	−0.168	(0.369)	0.161	(0.354)
Alcohol/Drug	16.876⁎⁎⁎	(0.000)	0.888	(0.103)	0.971	(0.243)	−0.436	(0.533)	0.339	(0.654)
Bi-polar disorder	18.819⁎⁎⁎	(0.000)	−0.033	(0.934)	−0.283	(0.504)	−0.454	(0.219)	−0.188	(0.599)
Personality disorder	0.463	(0.678)	0.147	(0.576)	−0.306	(0.322)	−0.094	(0.730)	−0.537⁎⁎	(0.037)
Drinking alcohol (Base: Never)										
Monthly or less	0.906	(0.240)	0.147	(0.122)	0.304⁎⁎⁎	(0.001)	−0.085	(0.340)	−0.014	(0.884)
2–4 times a month	0.081	(0.924)	−0.073	(0.429)	0.365⁎⁎⁎	(0.000)	−0.061	(0.495)	0.020	(0.832)
2–3 times a week	1.671*	(0.089)	0.040	(0.652)	0.410⁎⁎⁎	(0.000)	0.096	(0.266)	0.278⁎⁎⁎	(0.003)
More a week	1.561	(0.175)	−0.046	(0.648)	0.328⁎⁎⁎	(0.001)	−0.009	(0.930)	0.062	(0.569)
Using drugs	0.163	(0.891)	0.049	(0.774)	−0.357*	(0.070)	0.111	(0.588)	−0.176	(0.405)
Suicidal thoughts	0.000	(.)	0.174⁎⁎⁎	(0.003)	−0.317⁎⁎⁎	(0.000)	−0.233⁎⁎⁎	(0.000)	−0.148⁎⁎	(0.018)
Having mental health support from (Base: No)										
GP	−1.075	(0.207)	0.149*	(0.081)	0.195⁎⁎	(0.039)	−0.055	(0.567)	−0.012	(0.902)
Counsellor etc.	−1.340*	(0.079)	0.196	(0.108)	0.141	(0.289)	−0.059	(0.682)	0.089	(0.518)
Community Mental	0.679	(0.390)	0.303	(0.172)	−0.119	(0.561)	−0.424	(0.102)	−0.509⁎⁎	(0.016)
Psychiatric hospital	−0.000	(.)	0.000	(.)	0.000	(.)	0.000	(.)	0.000	(.)
Religious Leader	19.471⁎⁎⁎	(0.000)	−0.264	(0.419)	0.235	(0.525)	0.417	(0.129)	0.160	(0.678)
Constant1	39.318	(.)	−3.418⁎⁎⁎	(0.000)	−3.003⁎⁎⁎	(0.000)	−0.260	(0.572)	−2.155⁎⁎⁎	(0.000)
Constant2			−1.594⁎⁎⁎	(0.000)	−1.183⁎⁎⁎	(0.004)	0.513	(0.266)	0.535	(0.178)
Constant3			0.262	(0.538)	1.550⁎⁎⁎	(0.000)	1.369⁎⁎⁎	(0.003)	5.855⁎⁎⁎	(0.000)
Constant4			1.980⁎⁎⁎	(0.000)			1.560⁎⁎⁎	(0.001)		
Observations	152		5655		5655		5269		5655	
Adjusted *R*^2^	0.294		0.020		0.024		0.010		0.030	

*Note: p*-values are in parentheses.

⁎ *p* < 0.10.

⁎⁎ *p* < 0.05.

⁎⁎⁎ *p* < 0.01.

**Table 8:** Ordered logit regressions of ordered coping mechanism outcomes on healthcare professionals and other demographics.

	Good/coping activities changed		Amount of activity time impacted		Confident on coping	
	Coeff.	p-value	Coeff.	p-value	Coeff.	p-value
Health Professionals	0.028	(0.747)	−0.020	(0.781)	0.265⁎⁎⁎	(0.000)
Age Category (Base: below 21)						
21–24	0.175	(0.678)	0.969⁎⁎⁎	(0.008)	0.636*	(0.083)
25–34	0.335	(0.412)	0.885⁎⁎	(0.013)	0.887⁎⁎	(0.013)
35–44	0.070	(0.866)	0.904⁎⁎	(0.013)	0.826⁎⁎	(0.021)
45–54	−0.141	(0.732)	0.535	(0.142)	1.131⁎⁎⁎	(0.002)
55–64	−0.293	(0.479)	0.607*	(0.097)	1.276⁎⁎⁎	(0.000)
65 and over	−0.501	(0.272)	0.419	(0.312)	1.143⁎⁎⁎	(0.005)
Ethnicity (Base: others)						
White	0.022	(0.877)	−0.096	(0.419)	0.183	(0.107)
Asian	0.203	(0.478)	0.214	(0.325)	−0.186	(0.417)
Black	−0.175	(0.576)	−0.209	(0.554)	−0.203	(0.526)
Religion (Base: others)						
Christian	−0.053	(0.785)	0.197	(0.267)	−0.252	(0.132)
Muslim	0.131	(0.782)	0.299	(0.370)	0.104	(0.743)
Sikh	1.191	(0.254)	−0.201	(0.611)	−0.121	(0.867)
Hindu	−0.538	(0.187)	−0.096	(0.782)	−0.313	(0.328)
Jewish	−0.292	(0.579)	0.263	(0.515)	−0.317	(0.442)
Buddhist	−0.193	(0.589)	−0.359	(0.261)	0.481	(0.196)
No religion	−0.040	(0.836)	0.063	(0.722)	−0.131	(0.431)
Male	−0.071	(0.417)	−0.150⁎⁎	(0.039)	0.297⁎⁎⁎	(0.000)
Left education (Base: before age 16)						
At age 16	−0.021	(0.882)	−0.305⁎⁎	(0.021)	−0.133	(0.310)
At age 18	0.180	(0.206)	−0.155	(0.195)	−0.335⁎⁎⁎	(0.007)
Attended University	0.272⁎⁎	(0.031)	−0.042	(0.691)	−0.063	(0.566)
Accommodation (Base: others)						
Own home	0.419⁎⁎	(0.044)	−0.194	(0.250)	0.680⁎⁎⁎	(0.001)
Shared	0.345	(0.259)	−0.071	(0.773)	0.388	(0.151)
Private rented	0.068	(0.753)	−0.238	(0.180)	0.533⁎⁎	(0.014)
Parent's home	0.000	(.)	0.000	(.)	0.000	(.)
Own home	0.531⁎⁎	(0.036)	−0.155	(0.437)	0.342	(0.140)
Keyworker (Base: no)						
Health and Social	−0.304⁎⁎⁎	(0.003)	−0.267⁎⁎⁎	(0.001)	−0.001	(0.989)
Education	0.613⁎⁎⁎	(0.004)	0.087	(0.522)	−0.161	(0.221)
Key public services	0.253	(0.480)	−0.356	(0.172)	0.342	(0.143)
Local government	0.035	(0.888)	−0.005	(0.980)	0.068	(0.727)
Food	−0.678⁎⁎⁎	(0.006)	−0.343	(0.152)	−0.185	(0.393)
Public safety	−0.184	(0.693)	0.862	(0.258)	0.347	(0.630)
Transport	−0.501	(0.202)	−0.421	(0.330)	−0.046	(0.914)
Utilities	0.346	(0.316)	−0.319	(0.266)	−0.196	(0.434)
Pre-existing health condition (Base: none)						
Anxiety	−0.345⁎⁎⁎	(0.007)	0.160	(0.119)	−0.559⁎⁎⁎	(0.000)
Panic Attacks	−0.468*	(0.052)	0.552⁎⁎	(0.018)	−0.561⁎⁎⁎	(0.008)
Anorexia	−0.481	(0.450)	−0.328	(0.629)	0.187	(0.781)
Psychosis	11.709⁎⁎⁎	(0.000)	−0.543	(0.331)	2.267*	(0.090)
Depression	−0.360⁎⁎⁎	(0.001)	0.072	(0.392)	−0.466⁎⁎⁎	(0.000)
Bulimia	−0.389	(0.408)	−0.073	(0.838)	−0.131	(0.705)
Social phobia	−0.772⁎⁎	(0.023)	0.189	(0.580)	−0.856⁎⁎⁎	(0.004)
Attention deficit	−0.900*	(0.097)	−0.144	(0.764)	0.022	(0.967)
Obsessive	−0.322	(0.217)	−0.011	(0.955)	−0.577⁎⁎	(0.014)
Post-Traumatic	−0.610⁎⁎⁎	(0.003)	0.117	(0.491)	−0.409⁎⁎	(0.030)
Alcohol/Drug	−0.839	(0.343)	0.405	(0.416)	−1.447⁎⁎⁎	(0.000)
Bi-polar disorder	−0.761⁎⁎	(0.015)	0.548	(0.164)	−0.138	(0.711)
Personality disorder	−0.562*	(0.072)	0.589*	(0.077)	−0.925⁎⁎⁎	(0.003)
Drinking alcohol (Base: Never)						
Monthly or less	0.190*	(0.063)	−0.038	(0.694)	0.090	(0.359)
2–4 times a month	0.411⁎⁎⁎	(0.000)	0.137	(0.156)	0.162*	(0.092)
2–3 times a week	0.655⁎⁎⁎	(0.000)	0.253⁎⁎⁎	(0.006)	0.105	(0.263)
More a week	0.486⁎⁎⁎	(0.000)	0.316⁎⁎⁎	(0.002)	0.068	(0.536)
Using drugs	−0.403*	(0.053)	0.117	(0.546)	−0.025	(0.905)
Suicidal thoughts	−0.260⁎⁎⁎	(0.001)	0.145⁎⁎	(0.016)	−0.584⁎⁎⁎	(0.000)
Having mental health support from (Base: No)						
GP	0.061	(0.572)	−0.089	(0.325)	−0.171*	(0.062)
Counsellor etc.	0.142	(0.401)	−0.033	(0.817)	−0.389⁎⁎⁎	(0.005)
Community Mental	−0.031	(0.919)	0.219	(0.388)	−0.542⁎⁎	(0.018)
Psychiatric hospital	0.000	(.)	0.000	(.)	0.000	(.)
Religious Leader	0.828*	(0.066)	0.147	(0.682)	0.408	(0.173)
Constant1	−1.785⁎⁎⁎	(0.000)	−2.573⁎⁎⁎	(0.000)	−4.145⁎⁎⁎	(0.000)
Constant2	−0.639	(0.206)	−1.227⁎⁎⁎	(0.007)	−1.511⁎⁎⁎	(0.001)
Constant3			0.106	(0.813)	0.463	(0.301)
Constant4			1.150⁎⁎	(0.011)	2.521⁎⁎⁎	(0.000)
Observations	5655		5269		5308	
Adjusted *R*^2^	0.035		0.011		0.047	

*Note: p*-values are in parentheses.

⁎ *p* < 0.10.

⁎⁎ *p* < 0.05.

⁎⁎⁎ *p* < 0.01.

Table 5, Table 6, Table 7, Table 8 indicate that HCP have lower mental health scale scores than others. For example, being a HCP decreases log-odds ratio of severe anxiety (GAD-7) by 0.180, which is statistically significant at 5%. Below is a summary of the effects of all other demographics and pre-existing health conditions and wellbeing on those outcomes, on which statistically significant effects are seen:

1 Age: As age increases, following government advice, coping activities, amount of coping activity time, and confidence in coping rise, but risky activities, communications with family and scores on PHQ-9 and GAD-7 fall.
2 Ethnicity: Black and Asian minority ethnicities show lower mental health support than white and others. Black minority participants show lower drug use than others. Asian minority participants show much higher suicidal thoughts than others. Relationships with friends and family are lower among White but higher among Asians.
3 Religion: Jewish participants report less risky activities than others. Christian participants report higher GAD-7 scores than others. Muslim and Hindu participants report higher drug use than others. Jewish participants receive higher mental health support than others. Christian participants report more worries about coronavirus than other religious groups. Sikh, then Muslims and then Christians maintained higher relations with friends and family than others, and they also had higher coping activities than others.
4 Gender: Male participants follow government advice less than female participants, and they have lower PHQ-9, GAD-7, IES-R scores, alcohol consumption, mental health difficulties, worries about the virus, communications with friends and family, and activity time compared female counterparts. They have higher confidence in coping than females.
5 Education: According to the data, as education increases, conformation to government advice decreases; PHQ-9, alcohol consumption, relationship with friends and family increases; GAD-7 scores, worries about the virus, coping activities, activity time, and confidence in coping decreases.
6 Accommodation: Those who live in own home report higher compliance with government advice, coping activities, and confidence on coping and lower scores on PHQ-9, GAD-7, IES-R, and impact on mental health.
7 Key worker: Likelihood of following government advice decreases in key workers working in health and social care, key public services, and food services. Food workers report high likelihood of doing risky activities, and transport workers, report the opposite. Food workers also report higher scores of PHQ-9 and GAD-7 compared to others. The log likelihood of drinking alcohol increases among transport and utility workers. While the drug use of most of the key workers increases, the opposite is reported by utility workers. Most of the key workers have higher likelihood of suicidal thoughts and worries about coronavirus compared to others. Health and social care workers have less communication with friends and family, and therefore, their coping activities and time of those activities have been lower than others. Education workers have been able to manage higher relations with friends and family, and higher coping activities than others.
8 Pre-existing health conditions: Respondents with pre-existing anxiety and panic attacks are more likely to have higher scores on PHQ-9, GAD-7, IES-R scores than others, and their coping activities reduce and confidence on coping reduces too. Individuals with anorexia and psychosis have lower likelihood of doing risky activities than others, but patients with psychosis have lower likelihood of having higher scores of GAD-7 than others. Individuals with Bulimia, Bipolar disorder, and personality disorder report less likelihood of following government advice than others. Most of the individuals with pre-existing health conditions have higher scores of PHQ-9, GAD-7 and IES-R than others. Alcohol drinking increases among individuals with social phobia, attention deficit, and personality disorder. Drug use increases among respondents with Bipolar disorder and personality disorder. Suicidal thoughts increase amongst almost all individuals with pre-existing health conditions. However, coping activities and confidence on coping reduce among most of the individuals with pre-existing health conditions.
9 Alcohol: Higher alcohol intake before COVID-19 leads to lower compliance with government advice but higher scores of PHQ-9, GAD-7 and IES-R, and also higher coping activities, communications with friends and family and confidence on coping.
10 Drug use: Participants who report drug use have less likelihood of following government advice, communicating with friends and family, and coping activities, but higher likelihood of doing risky activities, with higher scores on PHQ-9 and GAD-7 compared to others.
11 Suicidal thoughts: Individuals with pre-COVID-19 suicidal thoughts show lower likelihood of following government advice, communications with friends and family, coping activities, confidence on coping, but higher likelihood of doing risky activities, with higher scores on PHQ-9, GAD-7, and IES-R.
12 Mental health support: Mental health support from GP, and counsellor largely increases with higher scores of PHQ-9, GAD-7, IES-R, but shows reduced confidence in coping.

### 9.8 Marginal effects

Table 9 shows marginal effects of all HCP, male HCP, and female HCP on outcomes. They are estimated from ordered logit models including all other covariates as shown in Table 5, Table 6, Table 7, Table 8. For male and female HCP, separate ordered logit models have been conducted. Marginal effects imply changes in probabilities. For example, in the case of GAD-7, if an individual is a HCP, the probability of having minimal anxiety increases by 0.039 (or, in other words, the chance of having minimal anxiety increases by 3.9 percentage points). Similarly, if an individual is a HCP, their chance of having mild, moderate, and severe anxiety decrease by 1.4, 1.2 and 1.2 percentage points, respectively. If the HCP is male, their chance of having mild, moderate, and severe anxiety decrease further by 6.5, 3.7 and 3.4 percentage points. In the case of female HCP, those decreases are not statistically significant. So, male non-HCP are more anxious than male HCP, but female non-HCP are not more or less anxious than female HCP. Other anxiety indicators, such as worries about corona virus and confidence about coping strategies show similar indication. Most of the outcomes show insignificant results.

**Table 9:** Marginal effects of healthcare professionals, male healthcare professionals, and female healthcare professionals on outcomes.

	Followed government advice				Did risky activities					
	Very few time	Some of the time	Several time	Most of the time	Rare	Very few time	Some of the time	Several time	Most of the time	
Healthcare professional	−0.000	−0.000	−0.002	0.003	−0.004	0.003	0.000	0.000	0.000	
	(0.841)	(0.841)	(0.841)	(0.841)	(0.653)	(0.653)	(0.659)	(0.669)	(0.660)	
Male	−0.005	−0.007	−0.038	0.050	−0.010	0.009	0.001	0.000	0.000	
	(0.256)	(0.281)	(0.253)	(0.254)	(0.647)	(0.633)	(0.781)	(.)	(.)	
Female	0.001	0.001	0.005	−0.006	−0.002	0.002	0.000	0.000	0.000	
	(0.688)	(0.689)	(0.688)	(0.688)	(0.809)	(0.809)	(0.811)	(0.816)	(0.811)	
	Patient health questionnaire (PHQ-9)					Generalized anxiety disorder (GAD-7)				
	Minimal	Mild	Moderate	Moderate Severe	Severe	Minimal	Mild	Moderate	Severe	

Healthcare professional	0.023	−0.003	−0.008	−0.006	−0.005	0.039⁎⁎	−0.014⁎⁎	−0.012⁎⁎	−0.012⁎⁎	
	(0.114)	(0.116)	(0.114)	(0.114)	(0.116)	(0.016)	(0.016)	(0.016)	(0.016)	
Male	0.094⁎⁎	−0.027⁎⁎	−0.030⁎⁎	−0.023⁎⁎	−0.014⁎⁎	0.136⁎⁎⁎	−0.065⁎⁎⁎	−0.037⁎⁎⁎	−0.034⁎⁎⁎	
	(0.023)	(0.024)	(0.026)	(0.028)	(0.033)	(0.001)	(0.001)	(0.002)	(0.003)	
Female	0.016	−0.002	−0.006	−0.005	−0.004	0.026	−0.009	−0.008	−0.009	
	(0.302)	(0.304)	(0.302)	(0.302)	(0.303)	(0.134)	(0.134)	(0.134)	(0.135)	
	Impact of events scale-revised (IES-R)				Drinking alcohol changed					
	None	PTSD may be concern	Probably PTSD diagnosis	High PTSD	Decreased	Unchanged	Increased			

Healthcare professional	0.011	−0.003	−0.002	−0.006	0.010	−0.006	−0.004			
	(0.467)	(0.468)	(0.468)	(0.467)	(0.511)	(0.511)	(0.511)			
Male	0.038	−0.013	−0.008	−0.017	0.019	0.009	−0.028			
	(0.294)	(0.496)	(.)	(0.285)	(0.529)	(0.532)	(0.529)			
Female	0.007	−0.002	−0.001	−0.004	−0.009	−0.008	0.017			
	(0.676)	(0.676)	(0.676)	(0.676)	(0.296)	(0.296)	(0.296)			
	Drug use changed			Mental health support changed			Mental health affected			
	Decreased	Unchanged	Increased	Decreased	Unchanged	Increased	No	Some of the time	Most of the time	All of the time

Healthcare professional	−0.077	−0.000	0.077	−0.016	0.003	0.013	−0.017	0.001	0.007	0.009
	(0.397)	(0.995)	(0.526)	(0.530)	(0.529)	(0.535)	(0.462)	(0.498)	(0.462)	(0.462)
Male				0.081	−0.019	−0.063	0.014	−0.002	−0.004	−0.008
				(0.689)	(0.692)	(0.699)	(0.882)	(0.881)	(0.881)	(0.882)
Female				−0.021	0.004	0.017	−0.014	0.001	0.006	0.008
				(0.414)	(0.421)	(0.420)	(0.557)	(0.602)	(0.558)	(0.558)
	Mental health changed			Suicidal thoughts changed	Worried about corona virus					
	Decreased	Unchanged	Increased		Not at all	A little bit	Moderately	Quite a bit	Extremely	

Healthcare professional	0.015	−0.011	−0.005	0.033	0.003*	0.011*	0.014*	−0.013*	−0.015*	
	(0.584)	(0.584)	(0.584)	(.)	(0.085)	(0.083)	(0.082)	(0.083)	(0.082)	
Male					0.011*	0.040*	0.028*	−0.045*	−0.034*	
					(0.085)	(0.067)	(0.066)	(0.066)	(0.066)	
Female	0.012	−0.009	−0.004	0.115	0.002	0.007	0.010	−0.008	−0.011	
	(0.678)	(0.678)	(0.678)	(.)	(0.266)	(0.264)	(0.264)	(0.264)	(0.264)	
	Communicated with friends/family				Relationships impacted					
	Not at all	Every few days	Daily	Several times a day	Isolated	No change	Feeling closer	Having more arguments	Talking more	

Healthcare professional	−0.005	−0.012	0.005	0.012	0.011	−0.000	−0.003	−0.001	−0.007	
	(0.186)	(0.187)	(0.187)	(0.187)	(0.507)	(0.512)	(0.507)	(0.507)	(0.507)	
Male	−0.026⁎⁎	−0.055⁎⁎	0.041⁎⁎	0.040⁎⁎	−0.014	−0.001	0.004	0.001	0.009	
	(0.039)	(0.042)	(0.040)	(0.044)	(0.743)	(0.748)	(0.743)	(0.743)	(0.742)	
Female	−0.002	−0.006	0.002	0.006	0.017	−0.001	−0.005	−0.001	−0.010	
	(0.548)	(0.548)	(0.548)	(0.548)	(0.353)	(0.363)	(0.353)	(0.355)	(0.353)	
	Did good/coping activities				Coping activities changed					
	Not at all	Every few days	Daily	Several times a day	Decreased	Unchanged	Increased			

Healthcare professional	−0.004	−0.014	0.018	0.001	−0.002	−0.003	0.005			
	(0.291)	(0.291)	(0.291)	(0.296)	(0.747)	(0.747)	(0.747)			
Male	−0.016	−0.041	0.054	0.003	−0.026	−0.037	0.063			
	(0.203)	(0.209)	(0.206)	(0.226)	(0.148)	(0.135)	(0.137)			
Female	−0.002	−0.009	0.011	0.000	0.004	0.004	−0.007			
	(0.546)	(0.546)	(0.546)	(0.547)	(0.664)	(0.665)	(0.664)			
	Amount of activity time impacted					Confident on coping				
	Not at all	A little	A moderate amount	A lot	A great deal	Not at all	A little bit	Moderately	Quite a bit	Extremely

Healthcare professional	0.001	0.002	0.002	−0.000	−0.004	−0.001⁎⁎⁎	−0.014⁎⁎⁎	−0.035⁎⁎⁎	0.003⁎⁎	0.048⁎⁎⁎
	(0.781)	(0.781)	(0.781)	(0.781)	(0.781)	(0.003)	(0.000)	(0.000)	(0.035)	(0.000)
Male	0.007	0.015	0.016	−0.004	−0.034	−0.004*	−0.030⁎⁎⁎	−0.092⁎⁎⁎	−0.025⁎⁎⁎	0.151⁎⁎⁎
	(0.385)	(0.370)	(0.370)	(0.377)	(0.372)	(0.075)	(0.001)	(0.000)	(0.006)	(0.000)
Female	0.000	0.001	0.001	−0.000	−0.002	−0.001⁎⁎	−0.009⁎⁎	−0.023⁎⁎	0.003⁎⁎	0.030⁎⁎
	(0.902)	(0.902)	(0.902)	(0.902)	(0.902)	(0.045)	(0.026)	(0.026)	(0.049)	(0.026)

*Note: p*-values are in parentheses.

⁎ *p* < 0.10.

⁎⁎ *p* < 0.05.

⁎⁎⁎ *p* < 0.01.

## 10 Discussion

This paper describes the protocol and results of the first three weeks of recruitment to the international survey: Psychological impact of COVID −19. The responses have increased with time as more organisations are joining the project. Our aim is to report periodic results as the temporal changes will be relevant considering changing government guidance that will impact on the population's responses and emotions. In the final report, using data from all participant countries, we aim to compare the cultural and political landscapes impacting on psychological response to the pandemic, thereby informing future global crisis.

The survey was launched in complete lockdown, but within 3 weeks of these results, there has been minor movement. Our survey in the first three weeks has recruited mainly HCP. This is possible because the survey is supported by NHS organisations. There is a predominance of females, which is not in accordance with the general population, but considering the majority of HCP; this may be understood as there is reported majority of females in the healthcare workforce (NHS Digital, 2019). The ethnicity of respondents is not representative of the general population, but this may be a reflection of the areas where the survey was implemented in the first three weeks. The data reports that age and gender have a correlation with following government advice. This finding resonates the results from Fancourt and colleagues’ study (Fancourt et al., 2020).

Vindegaard and Eriksen Benros (2020) conducted a systematic review of studies measuring psychiatric symptoms or morbidities associated with COVID-19 among infected patients and among non-infected groups, the latter divided into psychiatric patients, HCP and non-HCP. They included 43 studies, of which two papers evaluated patients with confirmed COVID-19 infection, and 41 the indirect effect of the pandemic (2 on patients with pre-existing psychiatric disorders, 20 on medical healthcare workers and 19 on the general public). 18 of the studies were case-control studies or compared to norms, while 25 of the studies had no control groups. They concluded that among healthcare workers depression/depressive symptoms, anxiety, psychological distress and poor sleep quality were increased. Regarding the public, one paper revealed lower psychological well-being compared to before COVID-19, while a longitudinal study found no difference in anxiety, depression or stress symptoms early in the pandemic compared to after four weeks. A variety of factors were associated with a higher risk of psychiatric symptoms and/or low psychological well-being of the public including female gender, front-line HCP, and poor self-rated health.

In our survey three week analysis, 32% participants reported experiencing suicidal thoughts pre-COVID-19, equally distributed between HCP and non-HCP. The findings of higher rates of mild depression and anxiety in HCP are similar to other studies. However, there are dissimilarities in the findings of gender. Notably, the majority of studies in the systematic review had small numbers, except for a couple who had comparable numbers and were conducted in Asia. Our sample and results with majority HCP in the UK may also be biased by the fact that in week 3 a large proportion of the participant organisations are mental health trusts. The timing of the survey meant that the initial influx of infections had just passed. This may have affected HCPs’ levels of distress.

As we continue recruitment to the study, the temporal relations in findings will be interesting as they will correspond with changes in the general landscape. We also aim to get better insights as international sites start recruiting. It is also worth noting that at the time of writing there are two events that could and will likely influence the Asian and Black ethnic group psychology – the publication of the Public Health England report (Public Health England, 2020) which states that Black and Asian Minority Ethnic groups have died at a higher proportion and once infected are more likely to die. Secondly, the ‘Black lives matter’ movement has sparked large gatherings in urban areas providing a secondary psychological effect and likely increased infections amongst these communities.

## 11 Strengths and limitations

The collaborative effort with 50 NHS organisations, universities and charities is a key strength of the study. This will allow a representative sample from a wider geography. The two-staged approach of implementing the survey again in six months will allow an analysis of change. The global aspect of the study will allow an evaluation of the cultural and political landscape influencing the differences in the psychological impact of the pandemic.

The survey has used a convenience sample and therefore relies on a self-selected group of respondents who choose to complete the questionnaire. As this is a participatory study, numbers will depend on people willing to participate. However, there has been a good response so far, and we anticipate a large sample size. Another limitation of the study is that there is no control with a number of outcomes. As we have a two-staged approach, there will be some potential to evaluate change over time.

## 12 Future research

The results presented in this paper are initial responses and are likely to change as the sample size increases. We hope to gain more recruitment from the general population, and a revised communication strategy will be instrumental based on the initial results. Temporal changes will be interesting to analyze as the participant demographics may change, and responses may change based on the political and government guidance. The possibility of delayed psychological impact of the pandemic due to economic and other changes or delayed realization of how individuals had been feeling during the height of the crisis is also a possibility. We will therefore also be able to see correlations to other events that may coincide with data collation, such as mass gatherings, PHE publications, or other future national or international events.

Future research will need to consider interventions to support individuals who report adverse psychological impact of the pandemic and its resultant restrictions. Improving populations’ resilience and preparedness to crisis will also need to be a priority.

## 13 Conclusion

Evidence has shown an adverse psychological impact of previous pandemics on the population, especially non-healthcare professional's wellbeing, not only during the pandemic but also in long term. Research should focus on identifying the need, preparing services, and determining the factors that enhance and build resilience.

## CRediT authorship contribution statement

**Shanaya Rathod:** Conceptualization, Methodology, Supervision, Validation, Writing - original draft, Writing - review & editing. **Saseendran Pallikadavath:** Conceptualization, Methodology, Funding acquisition, Writing - review & editing. **Allan H. Young:** Validation, Supervision, Writing - review & editing. **Lizi Graves:** Conceptualization, Methodology, Project administration, Writing - review & editing. **Mohammad Mahbubur Rahman:** Methodology, Formal analysis, Writing - review & editing. **Ashlea Brooks:** Validation, Project administration, Writing - review & editing. **Mustafa Soomro:** Methodology, Writing - review & editing. **Pranay Rathod:** Conceptualization, Methodology, Validation, Writing - review & editing. **Peter Phiri:** Conceptualization, Methodology, Validation, Writing - review & editing.

## Declaration of Competing Interest

None.

## Appendix Supplementary materials

Image, application 1
